# CoSOV1Net: A Cone- and Spatial-Opponent Primary Visual Cortex-Inspired Neural Network for Lightweight Salient Object Detection

**DOI:** 10.3390/s23146450

**Published:** 2023-07-17

**Authors:** Didier Ndayikengurukiye, Max Mignotte

**Affiliations:** Département d’Informatique et de Recherche Opérationnelle, Université de Montréal, Montreal, QC H3C 3J7, Canada; mignotte@iro.umontreal.ca

**Keywords:** lightweight salient object detection, salient object detection, object detection, lightweight neural network, color opponent, cone-opponent, double-opponent, vision sensing

## Abstract

Salient object-detection models attempt to mimic the human visual system’s ability to select relevant objects in images. To this end, the development of deep neural networks on high-end computers has recently achieved high performance. However, developing deep neural network models with the same performance for resource-limited vision sensors or mobile devices remains a challenge. In this work, we propose CoSOV1net, a novel lightweight salient object-detection neural network model, inspired by the cone- and spatial-opponent processes of the primary visual cortex (V1), which inextricably link color and shape in human color perception. Our proposed model is trained from scratch, without using backbones from image classification or other tasks. Experiments on the most widely used and challenging datasets for salient object detection show that CoSOV1Net achieves competitive performance (i.e., $F_{\beta }=0.931$ on the ECSSD dataset) with state-of-the-art salient object-detection models while having a low number of parameters (1.14 M), low FLOPS (1.4 G) and high FPS (211.2) on GPU (Nvidia GeForce RTX 3090 Ti) compared to the state of the art in lightweight or nonlightweight salient object-detection tasks. Thus, CoSOV1net has turned out to be a lightweight salient object-detection model that can be adapted to mobile environments and resource-constrained devices.

## 1. Introduction

The human visual system (HVS) has the ability to select and process relevant information from among the large amount that is received. This relevant information in an image is called salient objects [1]. Salient object-detection models in computer vision try to mimic this phenomenon by detecting and segmenting salient objects in images. This is an important task, given its many applications in computer vision, such as object tracking, recognition and detection [2], advertisement optimization [3], image/video compression [4], image correction [5], analysis of iconographic illustrations [6], image retrieval [7], aesthetic evaluation [8], image quality evaluation [9], image retargeting [10], image editing [11] and image collages [12], to name a few. Thus, it has been the subject of intensive research in recent years and is still being investigated [13]. Salient object-detection models generally fall into two categories, namely conventional and deep learning-based models, which differ by their feature extraction process. The former use hand-crafted features, while the latter use features learned from a neural network. Thanks to powerful representation learning methods, deep learning-based salient object-detection models have recently shown superior performance over conventional models [13,14]. The high performance of these models is undeniable; however, generally, they are also heavy if we consider their number of parameters and the amount of memory occupied, in addition to their high computational cost and slow detection speed. This makes these models less practical for resource-limited vision sensors or mobile devices that have many constraints on their memory and computational capabilities, as well as for real-time applications [15,16]. Hence, there is a need for lightweight salient object-detection models whose performance is comparable to state-of-the-art models, with the advantages of being deployed on resource-limited vision sensors or mobile devices and having a detection speed that allows them to be used in real-time applications. Existing lightweight salient object-detection models have used different methodologies, such as backbones from nonlightweight classification models [17,18], the imitation of primate hierarchical visual perception [19], human attention mechanisms [16,19], etc.

In this work, we propose an original approach for a new lightweight neural network model, namely CoSOV1Net, for salient object detection, that can therefore be adapted to mobile environments and resource-limited or -constrained devices, with the additional properties of being able to be trained from scratch without having to use backbones developed from image-classification tasks and having few parameters, but with comparable performance with state-of-the-art models.

Given that detecting salient objects is a capability of the human visual system and that a normal human visual system performs this quickly and correctly, we used images or scenes encoding mechanism research advances in neuroscience, especially for the early stage of the human visual system [20,21,22]. Our strategy in this model is therefore inspired by two neuroscience discoveries in human color perception, namely:

1. The color-opponent encoding in the early stage of the HVS (human visual system) [23,24,25,26];
2. The fact that color and pattern are linked inextricably in human color perception [20,27].

Inspired by these neuroscience discoveries, we propose a cone- and spatial-opponent primary visual cortex (CoSOV1) module that extracts features at the spatial level and between color channels at the same time to integrate color in the patterns. This process is applied first on opposing color pair channels two by two and then to grouped feature maps through our deep neural network. Thus, based on the CoSOV1 module, we build a novel lightweight encoder–decoder deep neural network for salient object detection: CoSOV1Net, which has only 1.14 M parameters but comparable performance with state-of-the-art salient object-detection models. CoSOV1Net predicts salient maps at a speed of 4.4 FPS on an Intel CPU, i7-11700F and 211.2 FPS on a Nvidia GeForce RTX 3090 Ti GPU for 384×384 images and it has a low FLOPS=1.4 G. Therefore, CoSOV1net is a lightweight salient object-detection model that can be adapted for mobile environments and limited-resource devices.

Our contribution is threefold:

- We propose a novel approach to extract features from opposing color pairs in a neural network to exploit the strength of the color-opponent principle from human color perception. This approach permits the acceleration of neural network learning;
- We propose a novel strategy to integrate color in patterns in a neural network by extracting features locally and between color channels at the same time in successively grouped feature maps, which results in a reduction in the number of parameters and the depth of the neural network, while keeping good performance;
- We propose—for the first time, to our knowledge—a novel lightweight salient object-detection neural network architecture based on the proposed approach for learning opposing color pairs along with the strategy of integrating color in patterns. This model has few parameters, but its performance is comparable to state-of-the-art methods.

The rest of this work is organized as follows: Section 2 presents some lightweight models related to this approach; Section 3 presents our proposed lightweight salient object-detection model; Section 4 describes the datasets used, evaluation metrics, our experimental results and the comparison of our model with state-of-the-art models; Section 5 discusses our results; Section 6 concludes this work.

## 2. Related Work

Many salient object-detection models have been proposed and most of the influential advances in image-based salient object detection have been reviewed by Gupta et al. [13]. Herein, we present some conventional models and lightweight neural network models related to this approach.

### 2.1. Lightweight Salient Object Detection

In recent years, lightweight salient object-detection models have been proposed with different strategies and architectures. Qin et al. [28] designed $U^{2}net$, a lightweight salient object-detection model with a two-level nested Unet [29] neural network able to capture more contextual information from different scales, thanks to the mixture of receptive fields of different sizes. Its advantages are threefold: first, it increases the depth of the whole architecture without increasing the computational cost; second, it is trained from scratch without using pretrained backbones, thus being able to keep feature maps high-resolution; third, it has high accuracy. Its disadvantage is its number of parameters. Other models are based on streamlined architecture to build lightweight deep neural networks. MobileNets [30,31] and ShuffleNets [32,33], along with their variants, are among the latter models. MobileNets [30] uses architecture based on depthwise separable convolution. ShuffleNets [32] uses architecture based on pointwise group convolution and channel shuffle, as well as depthwise convolution, to greatly reduce computational cost while maintaining accuracy. Their advantages are their computational cost, accuracy and speed, while their disadvantages are their number of parameters and their input resolution. Other authors have been inspired by primate or human visual system processes. Thus, Liu et al. [19] designed HVPNet, a lightweight salient object-detection network based on a hierarchical visual perception (HVP) module that mimics the primate visual cortex for hierarchical perception learning, whereas Liu et al. [16] were inspired by human perception attention mechanisms in designing SAMNet, another lightweight salient object-detection network, based on a stereoscopically attentive multiscale (SAM) module that adopts a stereoscopic attention mechanism for effective and efficient multiscale learning. Their advantages are their computational cost and accuracy, while their disadvantages are their number of parameters and their input resolution.

### 2.2. Color-Opponent Models

Color opponency, which is a human color perception propriety, has inspired many authors who have defined channels or feature maps to tackle their image-processing tasks. Frintrop et al. [34] used three opponent channels—RG, BY and *I*—to extract features for their salient object-detection model.

To extract features for salient object detection, Ndayikengurukiye and Mignotte [1] used nine (9) opponent channels for RGB color space (RR: red–red; RG: red–green; RB: red–blue; GR: green–red; GG: green–green; GB: green–blue; BR: blue–red; BG: blue–green; BB: blue–blue) with a nonlinear combination, thanks to the OCLTP (opponent color local ternary pattern) texture descriptor, which is an extension of the OCLBP (opponent color local binary pattern) [35,36] and Fastmap [37], which is a fast version of MDS (multidimensional scaling).

Most authors apply the opponent color mechanism to the input image color space channels and not on the resulting feature maps. However, Jain and Healey [38] used opponent features computed from Gabor filter outputs. They computed opponent features by combining information across different spectral bands at different scales obtained via Gabor filters for color texture recognition [38]. Yang et al. [39] proposed a framework based on the color-opponent mechanisms of color-sensitive double-opponent (DO) cells in the human visual system’s primary visual cortex (V1) in order to combine brightness and color to maximize the boundary-detection reliability in natural scenes. The advantages of hand-crafted models are their computational cost, number of parameters, speed and input resolution, while their disadvantage is accuracy.

In this work, we propose a model inspired by the human visual system but different from other models, because our model uses the primary visual cortex (V1) cone- and spatial-opponent principle to extract features at channels’ spatial levels and between color channels at the same time to integrate color into patterns in a manner allowing for a lightweight deep neural network design with performance comparable with state-of-the-art lightweight salient object-detection models.

## 3. Materials and Methods

### 3.1. Introduction

Our model for tackling the challenge of lightweight salient object detection is inspired by the human visual system (HVS)’s early visual color process, especially its cone opponency and spatial opponency in the primary visual cortex (V1). The human retina (located in the inner surface of the eye) has two types of photoreceptors, namely rods and cones. Rods are responsible for monochromatic vision under low levels of illumination, while cones are responsible for color vision at normal levels of illumination. There are three classes of cones: L, M and S. When light is absorbed by cone photoreceptors, the L, M and S cones absorb long-, middle- and short-wavelength visible light, respectively [24,25,27].

The cone signals are then processed by single-opponent retina ganglion cells. The single opponent operates an antagonistic comparison of the cone signals [23,25,26,40]:

- L − M opponent for red–green;
- S − (L + M) opponent for blue–yellow.

The red–green and blue–yellow signals are carried by specific cells (different cells each for red–green and blue–yellow) through the lateral geniculate nucleus (LGN) to the primary visual cortex (V1).

Shapley [27] and Shapley and Hawken [20] showed that the primary visual cortex (V1) plays an important role in color perception through the combined activity of two kinds of color-sensitive cortical neurons, namely single-opponent and double-opponent cells. Single-opponent cells in V1 operate in the same manner as those of retina ganglion cells and provide neuronal signals that can be used for estimating the color of the illumination [27]. Double-opponent cells in V1 compare cone signals across space as well as between cones [21,22,24,27]. Double-opponent cells thus have two opponencies: spatial opponency and cone opponency. These properties permit them to be sensitive to color edges and spatial patterns. They are thus able to inextricably link color and pattern in human color perception [20,27].

As the primary visual cortex (V1) is known to play a major role in visual color perception, as highlighted above, in this work, we propose a deep neural network based on the primary visual cortex (V1) to tackle the challenge of lightweight salient object detection. In particular, we use two neuroscience discoveries in human color perception, namely:

1. The color-opponent encoding in the early stage of the HVS;
2. The fact that color and pattern are inextricably linked in human color perception

These two discoveries in neuroscience inspired us to design a neural network architecture for lightweight salient object detection, which hinges on two main ideas. First, at the beginning of the neural network, our model opposes color channels two by two by grouping them (R-R, R-G, R-B, G-G, G-B, B-B) then extracting the features at the channels’ spatial levels and between the color channels from each channel pair at the same time, to integrate color into patterns. Therefore, instead of performing a subtractive comparison or an OCLTP (opponent color linear ternary pattern) like Ndayikengurukiye and Mignotte [1], we let the neural network learn the features that represent the comparison of the two color pairs. Second, this idea of grouping and then extracting the features at the channels’ spatial levels and between the color channels at the same time is applied on feature maps at each neural network level until the saliency maps are obtained. This process allows the proposed model to mimic the human visual system’s capability of inextricably linking color and pattern in color perception [20,27].

It is this idea that differentiates our model from other models that use depthwise convolution followed by pointwise convolution [30,31] to extract features at each individual color channel level (or feature map) first, not through a group of color channels (or feature maps) at the same time, as our model does. This idea also differentiates our model from models that combine a group of color channels (or feature maps) pixel by pixel first and apply depthwise convolution afterwards [32,33]. The idea of grouping color channels in pairs (or feature map groups) differentiates our model from models that consider all color channels (or feature maps) as a single group while extracting features at color channels’ spatial levels and between color channels at the same time.

Our model takes into account nonlinearities in the image at the beginning as well as through our neural network. For this purpose, we use an encoder–decoder neural network type whose core is a module that we call CoSOV1 (cone- and spatial-opponent primary visual cortex).

### 3.2. CoSOV1: Cone- and Spatial-Opponent Primary Visual Cortex Module

**Figure 1:**
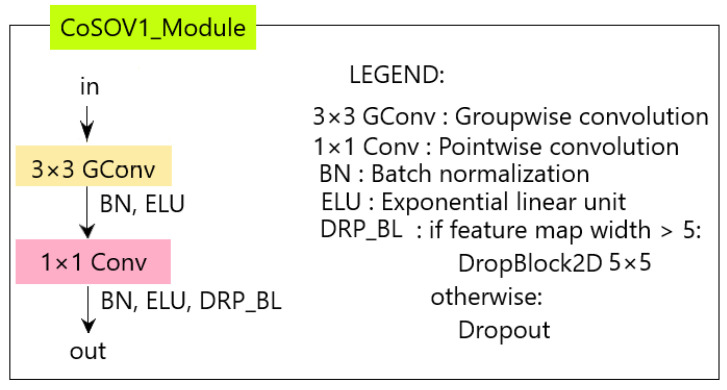
The CoSOV1 (cone- and spatial-opponent primary visual cortex) module is the core of our neural network model.

The CoSOV1 (cone- and spatial-opponent primary visual cortex) module is composed of two parts (see Figure 1).

In the first part, input color channels (or input feature maps) are split into groups of equal depth. Convolution (3×3) operations are then applied to each group of channels (or feature maps) in order to extract features from each group as opposing color channels (or opposing feature maps). This is performed thanks to a set of filters that convolve the group of color channels (or feature maps). Each filter is applied to the color channels (or input feature maps) through a convolution operation that detects local features at all locations on the input. Let $I^{g}\in R^{W\times H\times S}$ be an input group of feature maps, where W and H are the width and the height of each group’s feature map, respectively, and $W\in R^{3\times 3\times S}$, a filter with learned weights, with *S* being the depth of each group or the number of the channels in each group *g*, with g∈{1,…,G} (where G is the number of groups). The output feature map $O^{g}\in R^{W\times H}$ for this group *g* has a pixel value in the (k,l) position, defined as follows:

(1) $$O_{k,l}^{g}=\sum_{s=1}^{S}\sum_{i=0}^{2}\sum_{j=0}^{2}W_{i,j,s}I_{k+i-1,l+j-1,s}^{g}$$

The weight matrix $W\in R^{3\times 3\times S}$ is the same across the whole group of channels or feature maps. Therefore, each resulting output feature map represents a particular feature at all locations in the input color channels (or input feature maps) [41]. We call the 3×3 convolution on grouped channels (or grouped feature maps) groupwise convolution. The zero padding is applied during the convolution process to keep the input channel size for the output feature maps. After groupwise convolution, we apply the batch normalization transform, which is known to enable faster and more stable training of deep neural networks [42,43]. Let $B=\{X_{1},\ldots ,X_{K}\}$ be a minibatch that contains *K* examples from a dataset. The minibatch mean is

(2) $$\mu_{B}=\frac{1}{K}\sum_{k=1}^{K}X_{k}$$

and the minibatch variance is

(3) $$\sigma_{B}^{2}=\frac{1}{K}\sum_{k=1}^{K}{\left(X_{k}-\mu_{B}\right)}^{2}$$

The batch normalization transform $BN_{\gamma ,\beta }$: $\left\{X_{1},\ldots ,X_{K}\right\}⟶\left\{Y_{1},\ldots ,Y_{K}\right\}$ (γ and β are parameters to be learned):

(4) $$Y_{k}=\gamma \hat{X_{k}}+\beta$$

where k∈{1,…,K} and

(5) $$\hat{X_{k}}=\frac{X_{k}-\mu_{B}}{\sqrt{\sigma_{B}^{2}+\epsilon }}$$

and ϵ is a very small constant to avoid division by zero.

In order to take into account the nonlinearities present in the color channel input (or feature map input), given that groupwise convolution is a linear transformation, batch normalization is followed by a nonlinear function, exponential linear unit (ELU), defined as follows:

(6) $$\mathrm{ELU}\left(x\right)=\left\{\begin{array}{cc} x & \mathrm{if}\;x\geq 0,\\ \alpha \times (\exp(x)-1) & \mathrm{otherwise} \end{array}\right.$$

where α=1 by default.

The nonlinear function, which is the activation function, is placed after batch normalization, as recommended by Chollet [44].

The second part of the module searches for the best representation of the obtained feature maps. It is similar to the first part of the module, except for the groupwise convolution, which is replaced by point-wise convolution, but the input feature maps for pointwise convolution in this model are not grouped. Pointwise convolution allows us to learn the filters’ weights and thus obtain feature maps that best represent the input channels (or input feature maps) for the salient object-detection task, while having few parameters.

Let $O\in R^{W\times H\times M}$ be the output of the first part of the module, with *M* being the number of feature maps in this output and W and H being the width and the height, respectively. Let a filter of the learned weights $V\in R^{M}$ and $\mathrm{FM}\in R^{W\times H}$ be its output feature map by pointwise convolution. Its pixel value ${\mathrm{FM}}_{k,l}$ in (k,l) position is:

(7) $${\mathrm{FM}}_{k,l}=\sum_{m=1}^{M}V_{m}O_{k,l,m}$$

Thus, $V\in R^{M}$ is a vector of learned weights that associates the input feature maps $O\in R^{W\times H\times M}$ to the feature map $\mathrm{FM}\in R^{W\times H}$, which is the best representation of the latter-mentioned input feature maps. The pointwise convolution in this module uses many filters and thus it outputs many feature maps that are the best representation of the input feature map O. As pointwise convolution is a linear combination, we again apply batch normalization followed by a exponential linear unit function (ELU) on the feature map FM to obtain the best representation of the input feature maps for the learned weights $V\in R^{M}$, which takes into account nonlinearities in the feature maps $O\in R^{W\times H\times M}$.

Our scheme is different from depthwise separable convolution in that it uses the convolution of a group of channels instead of each channel individually [30,45].

In addition, after the nonlinear function, noise is injected in the resulting feature maps during the neural network learning stage thanks to the dropout process (but not in the prediction stage) to facilitate the learning process. In this model, we use DropBlock [46] if the width of the feature map is greater than 5; otherwise, we use the common dropout [47].

The CoSOV1 module allows our neural network to have few parameters but good performance.

### 3.3. CoSOV1Net Neural Network Model Architecture

**Figure 2:**
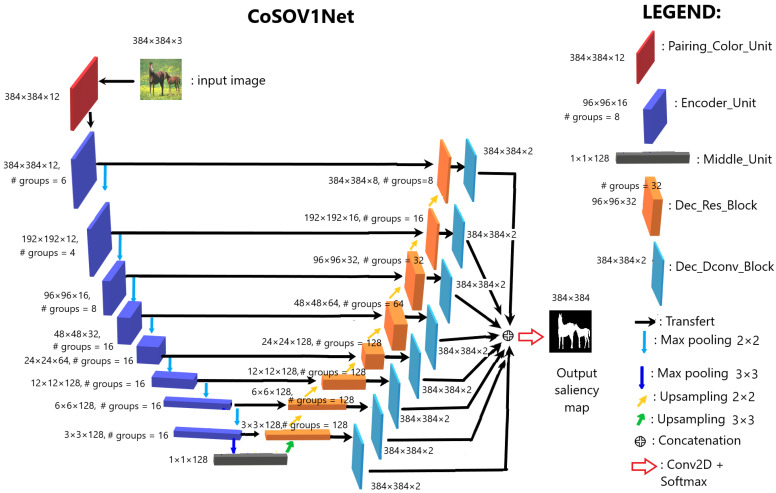
Our model CoSOV1 neural network architecture consisting of 5 blocks: Pairing_Color_Unit, Encoder_Unit, Middle_Unit, Dec_Res_Block and Dec_Dconv_Block.

Our proposed model is built on the CoSOV1 module (see Figure 1). It is a neural network of the U-net encoder–decoder type [29] and is illustrated in Figure 2. Thus, our model consists of three main blocks:

1. The input RGB color channel pairing;
2. The encoder;
3. The decoder.

#### 3.3.1. Input RGB Color Channel Pairing

**Figure 3:**
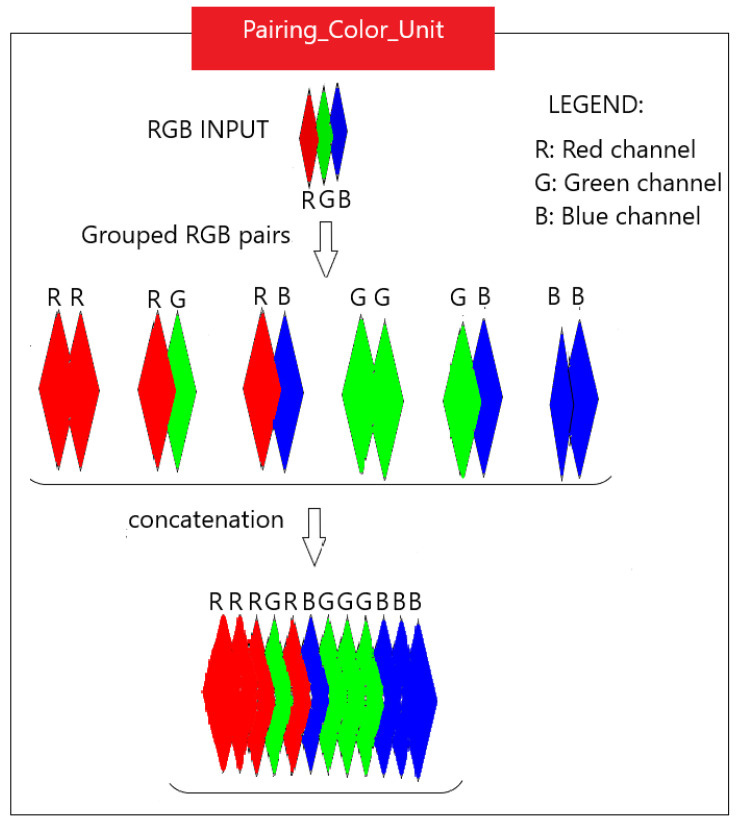
Pairing_Color_Unit: input RGB color image is transformed in 6 opposing color channel pairs; these are then concatenated to obtain 12 color channels.

At this stage, through Pairing_Color_Unit, the input RGB image is paired in six opposing color channel pairs: R-R, R-G, R-B, G-G, G-B and B-B [1,35,48]. These pairs are then concatenated, which gives 12 channels, R, R, R, G, R, B, G, G, G, B, B, B, as illustrated in Figure 3. This is the step for choosing the color channels to oppose. The set of concatenated color channels is then fed to the encoder.

#### 3.3.2. Encoder

The encoder, in our proposed neural network model, is a convolutional neural network (CNN) [49] where an encoder unit (see Figure 2) is repeated eight times. Each encoder unit is followed by a max pooling (2×2) with strides = 2, except for the eighth neural network level, where the max pooling is 3×3 with strides = 3 (the max pooling is a downsampling operation, like a filtering with a maximum filter). While the size of each feature map is reduced by half, the depth of the feature maps is doubled, except for the first level, where it is kept at 12 and the last two levels, where it is kept at 128 to have few parameters.

**Figure 4:**
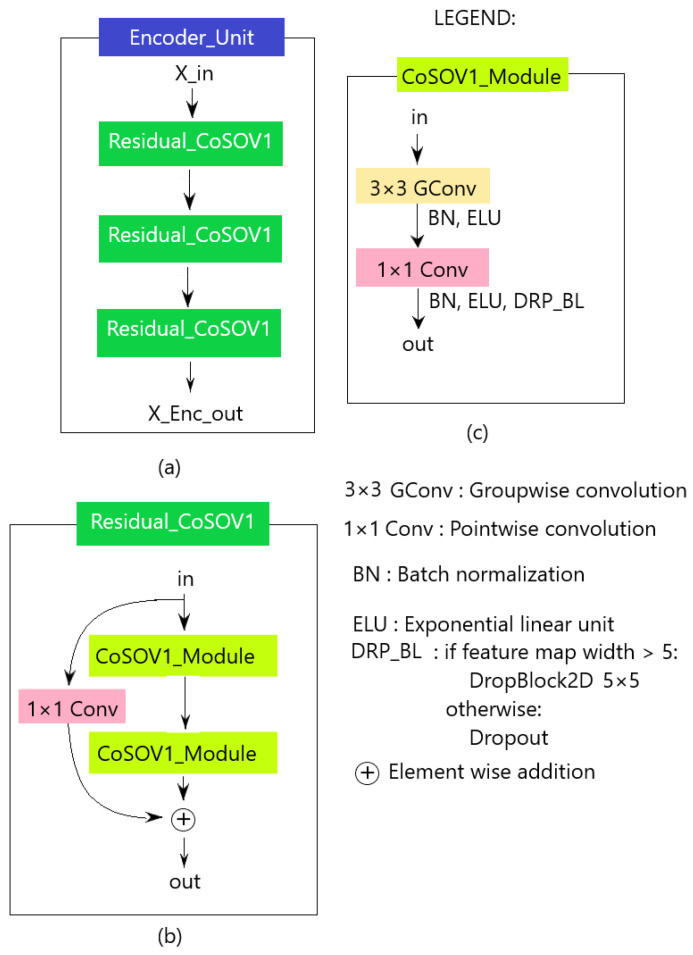
Encoder unit: (**a**) encoder unit; (**b**) the residual block; (**c**) CoSOV1 module.

The encoder unit (see Figure 4a) is composed of a residual block (Figure 4b) repeated three (3) times.

We used the residual block because this kind of block is known to improve the training of deeper neural networks [50]. The residual block consists of two CoSOV1 modules with a residual link. The reason for all these repetitions is to encode more information and thus allow our network performance to increase.

**Figure 5:**
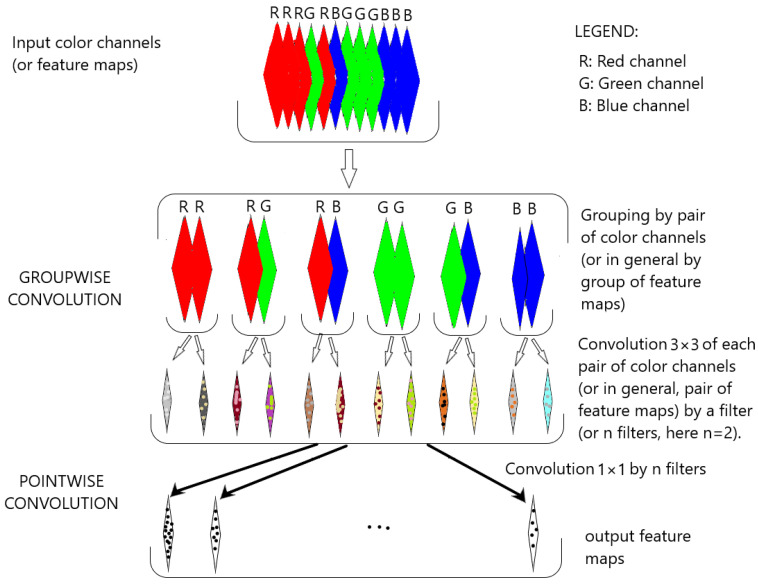
Simplified flowchart in CoSOV1 module for processing pairs of opposing color pairs (or group of feature maps).

In the encoder, schematically, as explained above (Section 3.2), the CoSOV1 module (Figure 4c) splits the input channels into groups and applies groupwise convolution (3×3 convolution). Then, pointwise convolution is applied to the outputs of the concatenated groups (see Figure 5 for the first-level input illustration). Each of these convolutions is followed by batch normalization and a nonlinear function (ELU: exponential linear unit activation). After these layers, during the model training, regularization is performed in the CoSOV1 module using the dropout [47] method for small feature maps (dimensions smaller than 5×5) and DropBlock [46]—which is a variant of dropout that zeroes a block instead of pixels individually as dropout does—for feature maps with dimensions greater than 5×5.

**Figure 6:**
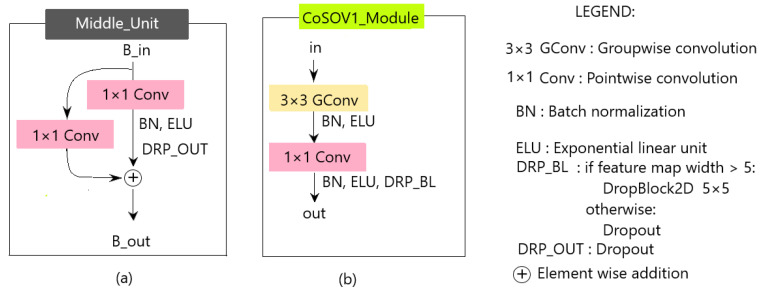
(**a**) The middle unit, (**b**) the CoSOV1 module.

At its end, the encoder is followed by the middle unit (see Figure 6a), which is the CoSOV1 module (see Figure 6b), where we remove the groupwise convolution—since at this stage, the feature maps are 1×1×128 in size—and add a residual link.

#### 3.3.3. Decoder

**Figure 7:**
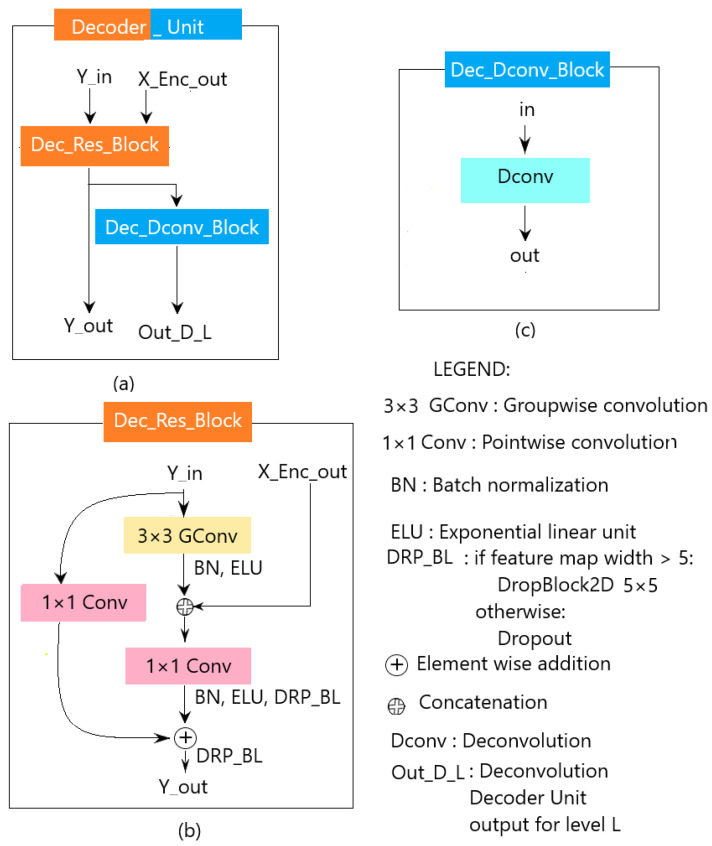
(**a**) The decoder unit; (**b**) the decoder residual block; (**c**) the decoder deconvolution block.

The decoder transforms the features from the encoder to obtain the estimate of the salient object(s) present in the input image. This transformation is achieved through a repeating block, namely the decoder unit (see Figure 7a). The decoder unit consists of two parts: the decoder residual block (see Figure 7b) and the decoder deconvolution block (see Figure 7c). The decoder residual block is a modified CoSOV1 module that allows the model to take into account the output of the corresponding level in the encoder. The output of the decoder residual block takes two directions. On the one hand, it is passed to the next level of the decoder; and on the other, to the second part of the decoder unit, which is the decoder deconvolution block. The latter deconvolves this output, obtaining two feature maps having the size of the input image (384×384×2 in our case). At the last level of the decoder, all the outputs from the deconvolution blocks are concatenated and fed to a convolution layer followed by a softmax activation layer, which gives the estimation of the salient object-detection map.

## 4. Experimental Results

### 4.1. Implementation Details

For our proposed model implementation, we used the deep learning platform TensorFlow with the Keras deep learning application programming interface (API) [51]. All input images were resized to 384×384 and pixel values were normalized (each pixel channel value ∈[0.0,…,1.0] and ground truth pixels ∈{0,1}). Experiments were conducted on a single GPU, Nvidia GeForce RTX 3090 Ti (24 GB) and an Intel CPU, i7-11700F.

### 4.2. Datasets

Our proposed model’s experiments were conducted on public datasets, which are the most widely used in the field of salient object detection [52]. Thus, we used the Extended Complex Scene Saliency dataset (ECSSD) [53] and the DUT-OMRON (Dalian University of Technology—OMRON Corporation) [54], DUTS [55], HKU-IS [56] and THUR15K [57] datasets.

ECSSD [53] contains 1000 natural images and their ground truths. Many of its images are semantically meaningful but structurally complex for saliency detection [53].

DUT-OMRON [54] contains 5168 images and their binary masks, with diverse variations and complex backgrounds.

The DUTS dataset [55] is divided into DUTS-TR (10,553 training images) and DUTS-TE (5019 test images). We trained and validated our proposed model on the DUTS-TR and DUTS-TE was used for tests.

HKU-IS [56] is composed of 4447 complex images, which contain many disconnected objects with different spatial distributions. Furthermore, it is very challenging for similar foreground/background appearances [58].

THUR15K is a dataset of images taken from the “Flickr” website, divided into five categories (butterfly, coffee mug, dog jump, giraffe, plane), which contains 3000 images. The images of this dataset represent real-world scenes and are considered complex for obtaining salient objects [57] (6232 images with ground truths).

### 4.3. Model Training Settings

For the reproducibility of the experiments, we set the seed = 123. We trained our proposed model on DUTS-TR (10,553 training images). We split the DUTS-TR dataset into a train set (9472 images) and a validation set (1056 images); that is, approximately 90% of the dataset for the training set and 10% for the validation set. We did not use 25 images because we wanted the training set and the validation set to be divisible by batch size, which is 32.

Our proposed model was trained on scratch without pretrained backbones from image classification (i.e., VGG [59], etc.) or lightweight backbones (i.e., MobileNets [30,31] or ShuffleNets [32,33]). As DUTS-TR is not a big dataset, we used data augmentation during training and many epochs in order to overcome this problem. Indeed, the more epochs, the more the data-augmentation process transforms data. Thus, our proposed model training has two successive stages:

- The first stage is with data augmentation, which is applied to each batch with random transformation (40% zoom in or horizontal flip or vertical flip). This stage has 480 epochs: 240 epochs with learning rate = 0.001 and 240 epochs with learning rate = 0.0001;
- The second stage is without data augmentation. It has 620 epochs: 240 epochs with learning rate = 0.001, followed by 140 epochs with learning rate = 0.0001 and 240 epochs with learning rate = 0.00005.

We also used the same initializer for all layers in the neural network: the HeUniform Keras initializer [60], which draws samples from a uniform distribution within [−limit, limit], where limit = $\sqrt{\frac{6}{fan\_in}}$ (fan_in is the number of input units in the weight tensor). The dropout rate was set to 0.2. We used the RMSprop [61] Keras optimizer with default values except for the learning rate; the centered, which was set to true; and the clipnorm = 1. The loss function used was the “sparse_categorical_crossentropy” Keras function; the Keras metric was “SparseCategoricalAccuracy; the Keras check point monitor was “val_sparse_categorical_accuracy”.

### 4.4. Hyperparameters

Hyperparameters such as the ELU activation function, the optimizer, the batch size, the filter size and the learning rates were chosen experimentally by observing the results.

The other hyperparameters were chosen as follows:

- Image size: The best image size was 384×384. We did not choose a small size because we expected to have a small salient object. As we also wanted to have a low computational cost, we did not go beyond this size.
- Number of levels for the encoder: We empirically obtained eight levels as the best number. The choice of image size permitted us to have a maximum of eight levels for the encoder part, given that $384=2^{7}\times 3$. The size of the feature maps of each level corresponds to the size of those of the previous level divided by 2, except the last level, where the division is by 3.
- Number of levels for the decoder: Eight levels. The number of levels is the same for the encoder part and the decoder part.
- Number of layers: At each level, we chose to use an encoder unit that has an equal number of layers for all levels and a decoder unit that has an equal number of layers for all levels. The number of layers was obtained experimentally.
- Number of filters: We also experimentally chose the number of filters keeping in mind the minimum parameters; the encoder’s number of filters was 12, 16, 32, 64, 128, 128, 128 and 128, respectively, for the first, second, *…*, seventh and eighth levels; the decoder residual bloc number of filters was 128, 128, 128, 128, 64, 32, 16 and 8, respectively, for the eighth, seventh, sixth, *…*, second and first levels. For the decoder deconvolution blocs, at each level, the number of filters was 2.
- The use of batch normalization: Batch normalization is known to enable faster and more stable training for deep neural networks [42,43]. So, we decided to use it.
- Use of dropout: The dropout process injects noise in the resulting feature maps during the neural network learning stage (but not in the prediction stage) to facilitate the learning process. In this model, we used DropBlock [46] if the width of the feature map was greater than 5; otherwise, we used the common dropout [47]. The best results were obtained for DropBlock size = 5×5 and rate = 0.1 (the authors’ paper suggested a value between 0.05 and 0.25). For the common dropout, the best rate was 0.2, obtained experimentally.

As our proposed model, CoSOV1Net does not use pretrained backbones and the input image is resized to 384×384; it has the advantage of good resolution.

### 4.5. Evaluation Metrics

#### 4.5.1. Accuracy

The metrics used to evaluate our proposed model accuracy were $F_{\beta }$ measure, MAE (mean absolute error) and weighted $F_{\beta }^{w}$ measure [62]. We also used precision, precision–recall and $F_{\beta }$ measure curves.

Let M be the binary mask obtained for the predicted saliency probability map, given a threshold in the range of [0,1) and with G being the corresponding ground truth:

(8) $$\begin{array}{c} \mathrm{Precision}=\frac{\left|M\cap G\right|}{\left|M\right|} \end{array}$$

(9) $$\begin{array}{c} \mathrm{Recall}=\frac{\left|M\cap G\right|}{\left|G\right|} \end{array}$$

∩: set intersection symbol; |.|: the number of pixels whose values are not zeros.

The $F_{\beta }$-measure ($F_{\beta }$) is the weighted harmonic mean of precision and recall:

(10) $$F_{\beta }=\frac{(1+\beta^{2})\times \mathrm{Precision}\times \mathrm{Recall}}{\beta^{2}\times \mathrm{Precision}+\mathrm{Recall}}$$

During evaluation, $\beta^{2}=0.3$, as it is often suggested [16,58].

Let $\bar{S}$ be the saliency map estimation with pixel values normalized in order to be in [0.0,…,1.0] and $\bar{G}$; its ground truth also normalized in {0;1}. The MAE (mean absolute error) is:

(11) $$MAE=\frac{1}{W\times H}\sum_{x=1}^{W}\sum_{y=1}^{H}\left|\bar{S}\left(x,y\right)-\bar{G}\left(x,y\right)\right|$$

where *W* and *H* are the width and the height, respectively, of the above maps ($\bar{S}$ and $\bar{G}$).

The $F_{\beta }^{w}$ measure [62] fixes the interpolation flaw, dependence flaw and equal importance flaw in traditional evaluation metrics and its value is:

(12) $$F_{\beta }^{w}=\left(1+\beta^{2}\right)\frac{{\mathrm{Precision}}^{w}\times {\mathrm{Recall}}^{w}}{\beta^{2}\times {\mathrm{Precision}}^{w}+{\mathrm{Recall}}^{w}}$$

${\mathrm{Precision}}^{w}$ and ${\mathrm{Recall}}^{w}$ are the weighted precision and the weighted recall, respectively.

#### 4.5.2. Lightweight Measures

Since we propose a lightweight salient object-detection model in this work, we therefore also evaluate the model with lightweight measures: the number of parameters, the saliency map estimation speed (FPS: frames per second) and the computational cost by measuring the FLOPS (the number of floating-point operations). The FLOPS is related to the device’s energy consumption (the higher the FLOPS, the higher the energy consumption). The floating-point operation numbers are computed as follows [63]:

- For a convolution layer with *n* filters of size k×k applied to W×H×C feature maps (W: width; H: height; C: channels), with P: number of parameters:
  (13) FLOPS=W×H×P
- For a max-pooling layer or an upsampling layer with a window of size sz×sz on W×H×C feature maps (*W*: width; *H*: height; *C*: channels):
  (14) FLOPS=W×H×C×sz×sz

### 4.6. Comparison with State of the Art

We compare our proposed model with 20 state-of-the-art salient object detection and 10 state-of-the-art lightweight salient object-detection models. We divided these methods because the lightweight methods outperform others with respect to lightweight measures. However, the lightweight methods’ accuracy is lower than the accuracy of those with huge parameters. We mainly used the salient object-detection results provided by Liu et al. [16], except for the $F_{\beta }$ measure and precision–recall curves, where we used saliency maps provided by these authors. We also used saliency maps provided by the HVPNet authors [19] to compute HVPNet $F_{\beta }^{\omega }$ measures.

In this section, we describe the comparison with the 20 salient object-detection models, namely DRFI [64], DCL [65], DHSNet [66], RFCN [67], NLDF [68], DSS [69], Amulet [18], UCF [70], SRM [71], PiCANet [17], BRN [72], C2S [73], RAS [74], DNA [75], CPD [76], BASNet [77], AFNet [78], PoolNet [79], EGNet [80] and BANet [81].

Table 1 shows that our proposed model CoSOV1Net outperforms all 20 state-of-the-art salient object-detection models for lightweight measures (#parameters, FLOPS and FPS) by a large margin (i.e., the best among them for FLOPS is DHSNet [66], with FLOPS=15.8 G and $F_{\beta }=0.903$ for ECSSD; the worst is EGNet [80], with FLOPS=270.8 G and $F_{\beta }=0.938$ for ECSSD; meanwhile, our proposed model, CoSOV1Net, has FLOPS=1.4 G, and its $F_{\beta }=0.931$ for ECSSD) (see Table 1).

Table 1 also shows that CoSOV1Net is among the top 6 models for ECSSD, among the top 7 for DUT-OMRON and around the top 10 for the other three datasets for the F-measure. Table 2 and Table 3 compare our model with the state-of-the-art models for the MAE and $F_{\beta }^{\omega }$ measures, respectively. From this comparison, we see that our model is ranked around the top 10 for all four datasets and is ranked 15th for the HKU-IS dataset. This demonstrates that our model is also competitive with respect to the performance of state-of-the-art models.

Table 1, Table 2 and Table 3 show that our proposed model, CoSOV1Net, clearly has the advantage of the number of parameters, computational cost and speed over salient object detection. They also show that its performance is closer to the best among them.

We also compared CoSOV1Net with the state-of-the-art lightweight salient object-detection models MobileNet [30], MobileNetV2 [31], ShuffleNet [32], ShuffleNetV2 [33], ICNet [82], BiSeNet R18 [83], BiSeNet X39 [83], DFANet [84], HVPNet [19] and SAMNet [16].

For the comparison with state-of-the-art lightweight models, Table 4 shows that our proposed model outperforms these state-of-the-art lightweight models in parameter numbers and the $F_{\beta }$ measure for the ECSSD dataset and is competitive for other measures and datasets. Table 5 shows that our model outperforms these state-of-the-art lightweight models for the MAE measure for the ECSSD and DUTS-TE datasets and is ranked first ex aequo with HVPNet for DUT-OMRON, first ex aequo with HVPNet and SAMNet for the HKU-IS dataset and second for the THUR15K dataset. Our model also outperforms these state-of-the-art lightweight models for the $F_{\beta }^{\omega }$ measure for ECSSD and DUTS-TE and is competitive for the three other datasets (see Table 6).

Table 4, Table 5 and Table 6 show that CoSOV1Net clearly has the advantage of the number of parameters over the lightweight salient object detection. They also show that its performance is closer to the best among them. Thus, CoSOV1Net has the advantage of performance.

Regarding computational cost, CoSOV1Net has an advantage over half of the state-of-the-art lightweight salient object-detection models. Overall, we can conclude that it has an advantage in terms of computational cost.

### 4.7. Comparison with SAMNet and HVPNet State of the Art

We chose to compare our CoSOV1Net model specifically with SAMNet [16] and HVPNet [19] because they are among the best state-of-the-art models.

**Figure 8:**
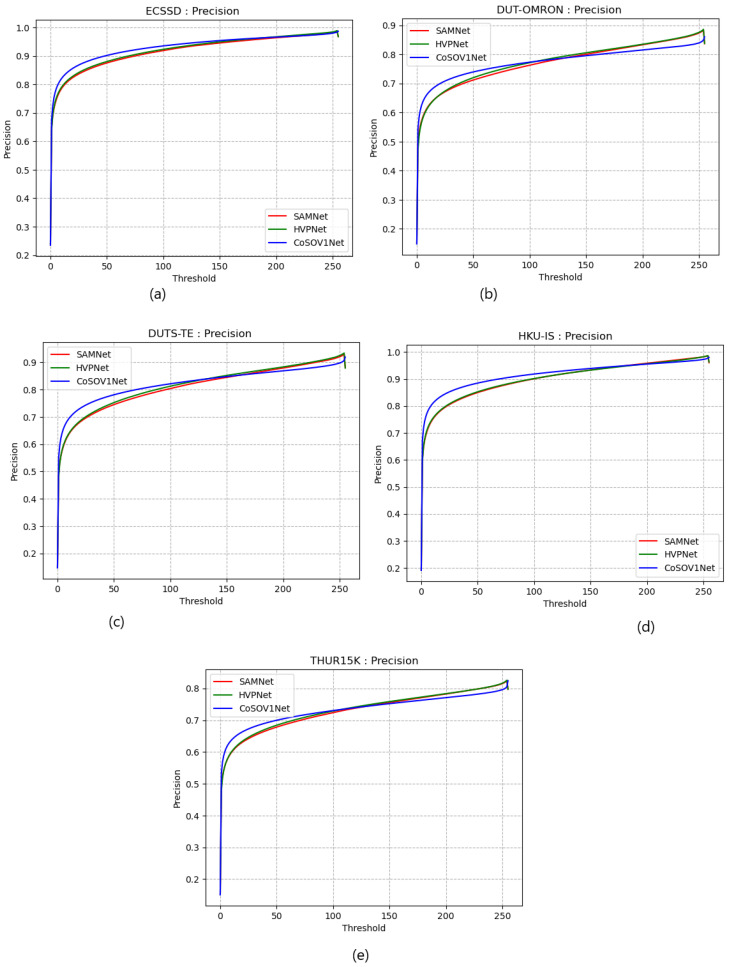
Precision curves for (**a**) ECSSD, (**b**) DUT-OMRON, (**c**) DUTS-TE, (**d**) HKU-IS and (**e**) THUR15K datasets.

Figure 8 shows that precision curves for ECSSD and HKU-IS datasets highlight that CoSOV1Net slightly dominates the SAMNet and HVPNet state-of-the-art lightweight salient object-detection models and that there is no clear domination for the DUT-OMRON, DUTS-TE and THUR15K precision curves between the three models. Therefore, the proposed model CoSOV1Net is competitive with these two state-of-the-art lightweight salient object-detection models with respect to precision.

**Figure 9:**
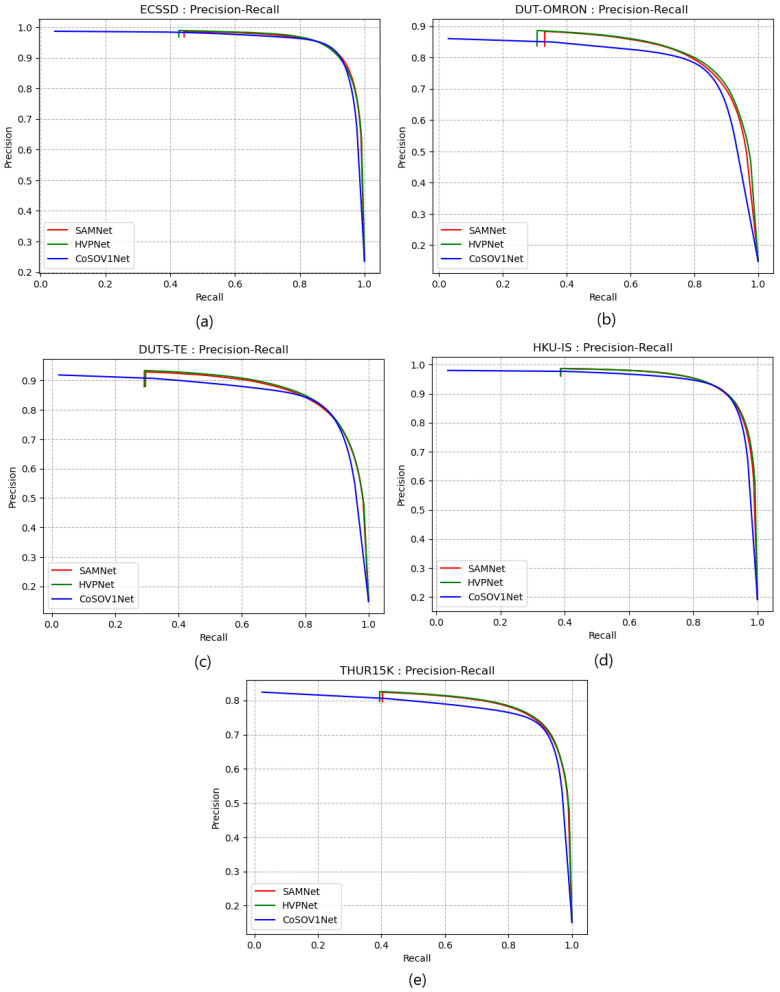
Precision–recall curves for (**a**) ECSSD, (**b**) DUT-OMRON, (**c**) DUTS-TE, (**d**) HKU-IS and (**e**) THUR15K datasets.

Figure 9 shows that the three models’ precision–recall curves (for the five datasets used: ECSSD, DUT-OMRON, DUTS-TE, HKU-IS and THUR15K) are very close to each other. Therefore, the proposed model is competitive with these two state-of-the-art lightweight salient object-detection models with respect to precision–recall.

**Figure 10:**
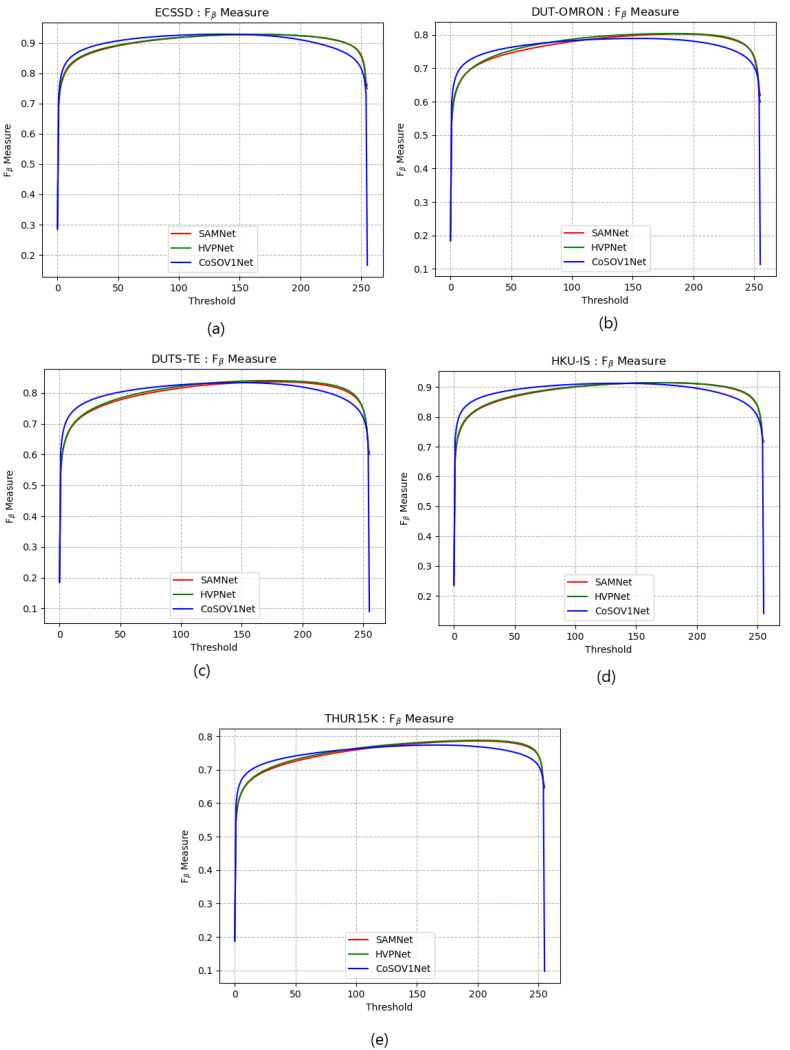
$F_{\beta }$ measure curves for (**a**) ECSSD, (**b**) DUT-OMRON, (**c**) DUTS-TE, (**d**) HKU-IS and (**e**) THUR15K datasets.

Figure 10 shows that the three models’ $F_{\beta }$ measure curves (for the five datasets used: ECSSD, DUT-OMRON, DUTS-TE, HKU-IS and THUR15K) are very close to each other. The CoSOV1Net model slightly dominates the two state-of-the-art lightweight salient object-detection models for thresholds ≤150 and the two state-of-the-art models slightly dominate for thresholds ≥150. Thus, there is no clear dominance for one model among the three. This proves that our CoSOV1Net model is comparable to these state-of-the-art lightweight salient object-detection models while having the advantage of a low number of parameters compared to them.

**Figure 11:**
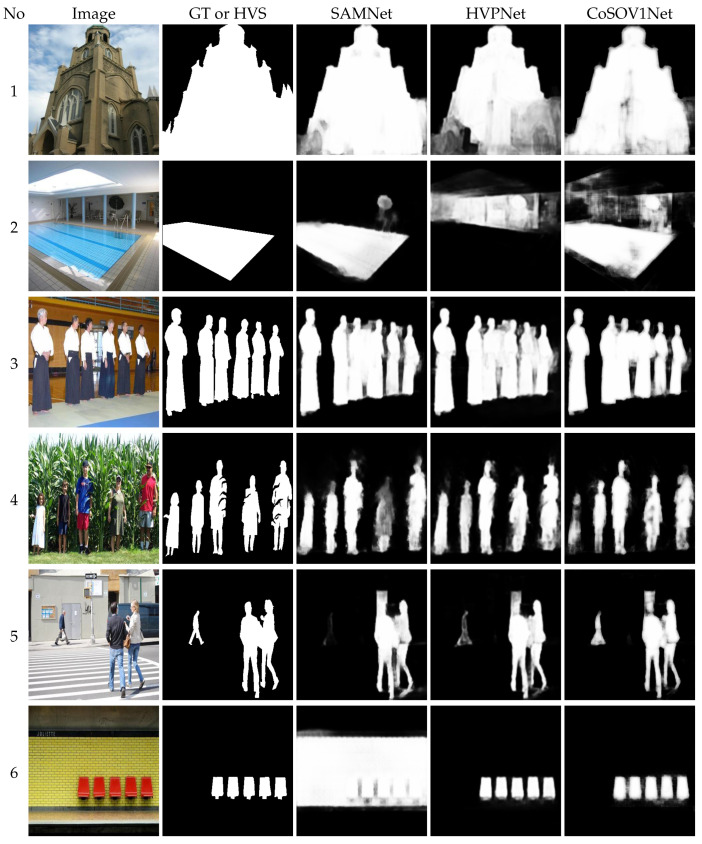

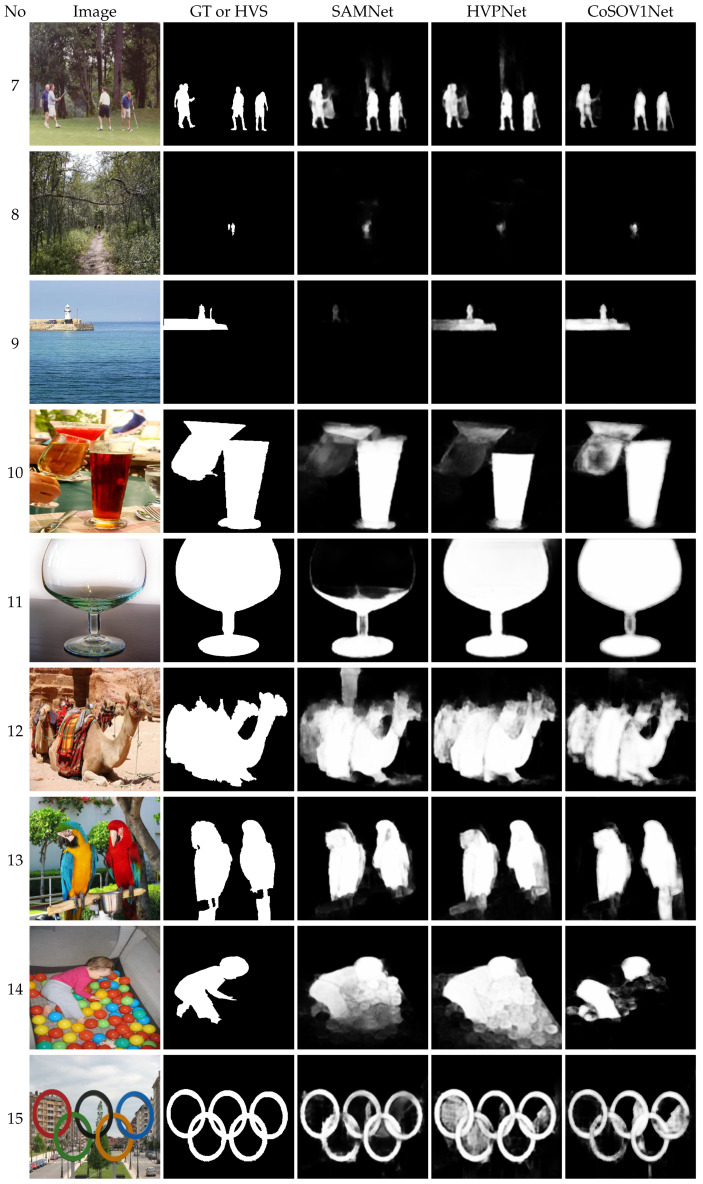

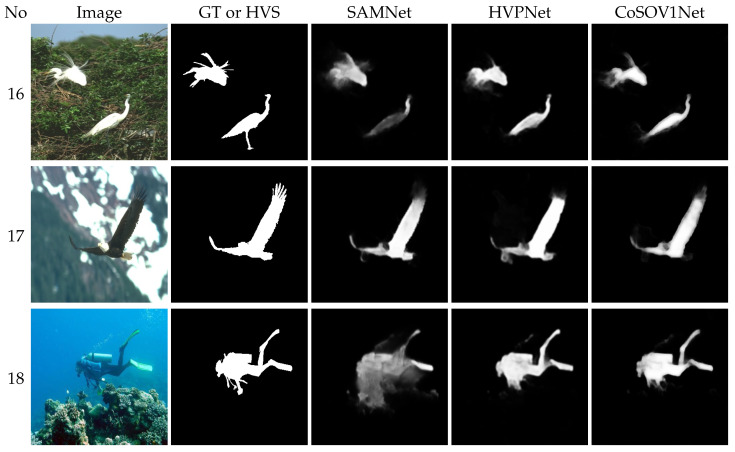
Comparison between SAMNet [16], HVPNet [19] and our proposed model, CoSOV1Net, on some image saliency maps: 1st column: images; 2nd column: ground truth or human visual system saliency map; 3rd column: SAMNet; 4th column: HVPNet; 5th column: CoSOV1Net (ours).

For qualitative comparison, Figure 11 shows some images highlighting that our proposed model (CoSOV1Net) is competitive with regard to the state-of-the-art SAMNet [16] and HVPNet [19] models, which are among the best ones.

Images from rows 1 and 2 show a big salient object on a cloudy background and a big object on a complex background, respectively: CoSOV1Net (ours) performs better than HVPNet on these saliency maps. Row 3 shows salient objects with the same colors and row 4 shows salient objects with multiple colors: the SAMNet and CoSOV1Net saliency maps are slightly identical and the HVPNet saliency map is slightly better. Row 5 shows n image with three salient objects with different sizes and colors: two are big and one is very small; the CoSOV1Net saliency map is better than SAMNet’s and HVPNet’s. Row 6 shows red salient objects on a black and yellow background; SAMNet’s saliency map is the worst, while CoSOV1Net and HVPNet perform well on that image. Row 7 shows a complex background and multiple salient objects with different colors: CoSOV1Net performs better than SAMNet and HVPNet. Row 8 shows tiny salient objects: the three models perform well. On row 9, SAMNet has the worst performance, while CoSOV1Net is the best. Row 10 shows colored glasses as salient objects: the CoSOV1Net performance is better than SAMNet’s and HVPNet’s. On row 11, SAMNet has the worst performance. On row 12 and 13, CoSOV1Net has the best performance. Row 18 shows a submarine image: CoSOV1Net is better than SAMNet.

Figure 8, Figure 9, Figure 10 and Figure 11 confirm that CoSOV1Net has an advantage on performance.

## 5. Discussion

The results show the performance of our model, CoSOV1Net, for accuracy measures and lightweight measures. CoSOV1Net’s rank, when compared to state-of-the-art models, shows that it behaves as a lightweight salient object-detection model by dominating lightweight measures and having good performance for accuracy measures (see Table 7).

The results also show that when CoSOV1Net is compared to state-of-the-art lightweight salient object-detection models, its measure results are generally ranked among the best for the datasets and measures used (see Table 8). Thus, we can conclude that CoSOV1Net behaves as a competitive lightweight salient object-detection model.

As we did not use backbones from image classification (i.e., VGG [59], *…*) or lightweight backbones (i.e., MobileNets [30,31] or ShuffleNets [32,33]), we conclude that CoSOV1Net’s performance is intrinsic to this model itself.

**Figure 12:**
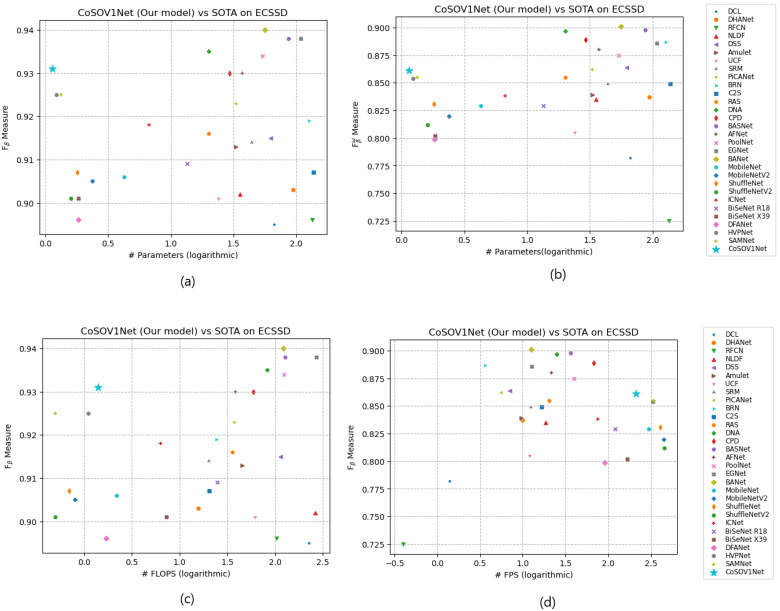
Example of trade-off between (**a**) $F_{\beta }$ measure and #parameters; (**b**) $F_{\beta }^{\omega }$ measure and #parameters; (**c**) $F_{\beta }$ measure and FLOPS; (**d**) $F_{\beta }$ measure and FPS, for ECSSD.

Finally, putting together the measures for salient object-detection models and lightweight salient object-detection models in a graphic, we noticed that the CoSOV1Net model is located for $F_{\beta }$ measures with respect to FLOPS and for the number of parameters in the top left, while for the FPS measure, it is located in the top right, thus demonstrating its performance as a lightweight salient object-detection model (see Figure 12). This shows that CoSOV1Net is competitive with the best state-of-the-art models used.

The quantitative and the qualitative comparisons with SAMNet [16] and HVPNet [19] showed that our proposed model has good performance, given that these state-of-the-art models are among the best ones.

## 6. Conclusions

In this work, we present a lightweight salient object-detection deep neural network, CoSOV1Net, with a very low number of parameters (1.14 M), a low floating-point operations number (FLOPS = 1.4 G) and thus low computational cost and respectable speed (FPS=211.2 on GPU: Nvidia GeForce RTX 3090 Ti), yet with comparable performance with state-of-the-art salient object-detection models that use significantly more parameters, and other lightweight salient object-detection models such as SAMNet [16] and HVPNet [19].

The novelty of our proposed model (CoSOV1Net) is that it uses the principle of integrating color in pattern in a salient object-detection deep neural network, since according to Shapley [27] and Shapley and Hawken [20], color and pattern are inextricably linked in color human perception. This is implemented by taking inspiration from the primary visual cortex (V1) cells, especially cone- and spatial-opponent cells. Thus, our method extracts features at the color channels’ spatial level and between the color channels at the same time on a pair of opposing color channels. The idea of grouping color pushed us to group feature maps through the neural network and extract features at the spatial level and between feature maps, as carried out for color channels.

Our results showed that this strategy generates a model that is very promising, competitive with most state-of-the-art salient object-detection and lightweight salient object-detection models and practical for mobile environments and limited-resource devices.

In future work, our proposed CoSOV1Net model, based on integrating color into patterns, can be improved by coupling it with the human visual system attention mechanism, which is the basis of many lightweight models, to tackle its speed limitation and thus produce a more efficient lightweight salient object-detection model.

## Figures and Tables

**Table 1:** Our proposed model F-measure ($F_{\beta }↑$, $\beta^{2}=0.3$) compared with 20 state-of-the-art models (best value in bold) [# Param: number of parameters, ↑: great is best, ↓: small is the best].

Methods	# Param (M) ↓	FLOPS (G) ↓	Speed (FPS) ↑	ECSSD	DUT-OMRON	DUTS-TE	HKU-IS	THUR15K
DRFI [64]	-	-	0.1	0.777	0.652	0.649	0.774	0.670
DCL [65]	66.24	224.9	1.4	0.895	0.733	0.785	0.892	0.747
DHSNet [66]	94.04	15.8	10.0	0.903	-	0.807	0.889	0.752
RFCN [67]	134.69	102.8	0.4	0.896	0.738	0.782	0.892	0.754
NLDF [68]	35.49	263.9	18.5	0.902	0.753	0.806	0.902	0.762
DSS [69]	62.23	114.6	7.0	0.915	0.774	0.827	0.913	0.770
Amulet [18]	33.15	45.3	9.7	0.913	0.743	0.778	0.897	0.755
UCF [70]	23.98	61.4	12.0	0.901	0.730	0.772	0.888	0.758
SRM [71]	43.74	20.3	12.3	0.914	0.769	0.826	0.906	0.778
PiCANet [17]	32.85	37.1	5.6	0.923	0.766	0.837	0.916	0.783
BRN [72]	126.35	24.1	3.6	0.919	0.774	0.827	0.910	0.769
C2S [73]	137.03	20.5	16.7	0.907	0.759	0.811	0.898	0.775
RAS [74]	20.13	35.6	20.4	0.916	0.785	0.831	0.913	0.772
DNA [75]	20.06	82.5	25.0	0.935	0.799	0.865	0.930	0.793
CPD [76]	29.23	59.5	68.0	0.930	0.794	0.861	0.924	0.795
BASNet [77]	87.06	127.3	36.2	0.938	**0.805**	0.859	0.928	0.783
AFNet [78]	37.11	38.4	21.6	0.930	0.784	0.857	0.921	0.791
PoolNet [79]	53.63	123.4	39.7	0.934	0.791	0.866	0.925	**0.800**
EGNet [80]	108.07	270.8	12.7	0.938	0.794	0.870	0.928	**0.800**
BANet [81]	55.90	121.6	12.5	**0.940**	0.803	**0.872**	**0.932**	0.796
CoSOV1Net (OURS)	**1.14**	**1.4**	**211.2**	0.931	0.789	0.833	0.912	0.773

**Table 2:** Our proposed model MAE (↓) compared with 20 state-of-the-art models (best performance in bold) [# Param: number of parameters, ↑: great is the best, ↓: small is the best].

Methods	# Param (M) ↓	FLOPS (G) ↓	Speed (FPS) ↑	ECSSD	DUT-OMRON	DUTS-TE	HKU-IS	THUR15K
DRFI [64]	-	-	0.1	0.161	0.138	0.154	0.146	0.150
DCL [65]	66.24	224.9	1.4	0.080	0.095	0.082	0.063	0.096
DHSNet [66]	94.04	15.8	10.0	0.062	-	0.066	0.053	0.082
RFCN [67]	134.69	102.8	0.4	0.097	0.095	0.089	0.080	0.100
NLDF [68]	35.49	263.9	18.5	0.066	0.080	0.065	0.048	0.080
DSS [69]	62.23	114.6	7.0	0.056	0.066	0.056	0.041	0.074
Amulet [18]	33.15	45.3	9.7	0.061	0.098	0.085	0.051	0.094
UCF [70]	23.98	61.4	12.0	0.071	0.120	0.112	0.062	0.112
SRM [71]	43.74	20.3	12.3	0.056	0.069	0.059	0.046	0.077
PiCANet [17]	32.85	37.1	5.6	0.049	0.068	0.054	0.042	0.083
BRN [72]	126.35	24.1	3.6	0.043	0.062	0.050	0.036	0.076
C2S [73]	137.03	20.5	16.7	0.057	0.072	0.062	0.046	0.083
RAS [74]	20.13	35.6	20.4	0.058	0.063	0.059	0.045	0.075
DNA [75]	20.06	82.5	25.0	0.041	**0.056**	0.044	**0.031**	0.069
CPD [76]	29.23	59.5	68.0	0.044	0.057	0.043	0.033	**0.068**
BASNet [77]	87.06	127.3	36.2	0.040	**0.056**	0.048	0.032	0.073
AFNet [78]	37.11	38.4	21.6	0.045	0.057	0.046	0.036	0.072
PoolNet [79]	53.63	123.4	39.7	0.048	0.057	0.043	0.037	**0.068**
EGNet [80]	108.07	270.8	12.7	0.044	**0.056**	0.044	0.034	0.070
BANet [81]	55.90	121.6	12.5	**0.038**	0.059	**0.040**	**0.031**	**0.068**
CoSOV1Net (OURS)	**1.14**	**1.4**	**211.2**	0.051	0.064	0.057	0.045	0.076

**Table 3:** Our proposed model weighted F-measure ($F_{\beta }^{\omega }↑$, $\beta^{2}=1$) compared with 20 state-of-the-art models (best value in bold) [# Param: number of parameters, ↑: great is the best, ↓: small is the best].

Methods	# Param (M) ↓	FLOPS (G) ↓	Speed (FPS) ↑	ECSSD	DUT-OMRON	DUTS-TE	HKU-IS	THUR15K
DRFI [64]	-	-	0.1	0.548	0.424	0.378	0.504	0.444
DCL [65]	66.24	224.9	1.4	0.782	0.584	0.632	0.770	0.624
DHSNet [66]	94.04	15.8	10.0	0.837	-	0.705	0.816	0.666
RFCN [67]	134.69	102.8	0.4	0.725	0.562	0.586	0.707	0.591
NLDF [68]	35.49	263.9	18.5	0.835	0.634	0.710	0.838	0.676
DSS [69]	62.23	114.6	7.0	0.864	0.688	0.752	0.862	0.702
Amulet [18]	33.15	45.3	9.7	0.839	0.626	0.657	0.817	0.650
UCF [70]	23.98	61.4	12.0	0.805	0.573	0.595	0.779	0.613
SRM [71]	43.74	20.3	12.3	0.849	0.658	0.721	0.835	0.684
PiCANet [17]	32.85	37.1	5.6	0.862	0.691	0.745	0.847	0.687
BRN [72]	126.35	24.1	3.6	0.887	0.709	0.774	0.875	0.712
C2S [73]	137.03	20.5	16.7	0.849	0.663	0.717	0.835	0.685
RAS [74]	20.13	35.6	20.4	0.855	0.695	0.739	0.849	0.691
DNA [75]	20.06	82.5	25.0	0.897	0.729	0.797	**0.889**	0.723
CPD [76]	29.23	59.5	68.0	0.889	0.715	0.799	0.879	**0.731**
BASNet [77]	87.06	127.3	36.2	0.898	**0.751**	0.802	**0.889**	0.721
AFNet [78]	37.11	38.4	21.6	0.880	0.717	0.784	0.869	0.719
PoolNet [79]	53.63	123.4	39.7	0.875	0.710	0.783	0.864	0.724
EGNet [80]	108.07	270.8	12.7	0.886	0.727	0.796	0.876	0.727
BANet [81]	55.90	121.6	12.5	**0.901**	0.736	**0.810**	**0.889**	0.730
CoSOV1Net (OURS)	**1.14**	**1.4**	**211.2**	0.861	0.696	0.731	0.834	0.688

**Table 4:** Our proposed model’s F-measure ($F_{\beta }↑$, $\beta^{2}=0.3$) compared with state-of-the-art lightweight salient object-detection models (best value in bold) [# Param: number of parameters, ↑: great is the best, ↓: small is the best].

Methods	# Param (M) ↓	FLOPS (G) ↓	Speed (FPS) ↑	ECSSD	DUT-OMRON	DUTS-TE	HKU-IS	THUR15K
MobileNet * [30]	4.27	2.2	295.8	0.906	0.753	0.804	0.895	0.767
MobileNetV2 * [31]	2.37	0.8	446.2	0.905	0.758	0.798	0.890	0.766
ShuffleNet * [32]	1.80	0.7	406.9	0.907	0.757	0.811	0.898	0.771
ShuffleNetV2 * [33]	1.60	**0.5**	**452.5**	0.901	0.746	0.789	0.884	0.755
ICNet [82]	6.70	6.3	75.1	0.918	0.773	0.810	0.898	0.768
BiSeNet R18 [83]	13.48	25.0	120.5	0.909	0.757	0.815	0.902	0.776
BiSeNet X39 [83]	1.84	7.3	165.8	0.901	0.755	0.787	0.888	0.756
DFANet [84]	1.83	1.7	91.4	0.896	0.750	0.791	0.884	0.757
HVPNet [19]	1.23	1.1	333.2	0.925	**0.799**	**0.839**	**0.915**	**0.787**
SAMNet [16]	1.33	**0.5**	343.2	0.925	0.797	0.835	**0.915**	0.785
CoSOV1Net (OURS)	**1.14**	1.4	211.2	**0.931**	0.789	0.833	0.912	0.773

* SAMNet, where the encoder is replaced by this backbone.

**Table 5:** Our proposed model MAE (↓) compared with state-of-the art lightweight salient object-detection models (best value in bold) [# Param: number of parameters, ↑: great is the best, ↓: small is the best].

Methods	# Param (M) ↓	FLOPS (G) ↓	Speed (FPS) ↑	ECSSD	DUT-OMRON	DUTS-TE	HKU-IS	THUR15K
MobileNet * [30]	4.27	2.2	295.8	0.064	0.073	0.066	0.052	0.081
MobileNetV2 * [31]	2.37	0.8	446.2	0.066	0.075	0.070	0.056	0.085
ShuffleNet * [32]	1.80	0.7	406.9	0.062	0.069	0.062	0.050	0.078
ShuffleNetV2 * [33]	1.60	**0.5**	**452.5**	0.069	0.076	0.071	0.059	0.086
ICNet [82]	6.70	6.3	75.1	0.059	0.072	0.067	0.052	0.084
BiSeNet R18 [83]	13.48	25.0	120.5	0.062	0.072	0.062	0.049	0.080
BiSeNet X39 [83]	1.84	7.3	165.8	0.070	0.078	0.074	0.059	0.090
DFANet [84]	1.83	1.7	91.4	0.073	0.078	0.075	0.061	0.089
HVPNet [19]	1.23	1.1	333.2	0.055	**0.064**	0.058	**0.045**	0.076
SAMNet [16]	1.33	**0.5**	343.2	0.053	0.065	0.058	**0.045**	**0.077**
CoSOV1Net (OURS)	**1.14**	1.4	211.2	**0.051**	**0.064**	**0.057**	**0.045**	0.076

* SAMNet, where the encoder is replaced by this backbone.

**Table 6:** Our proposed model’s weighted F-measure ($F_{\beta }^{\omega }↑$, $\beta^{2}=1$) compared with lightweight salient object-detection models (best value in bold) [# Param: number of parameters, ↑: great is the best, ↓: small is the best].

Methods	# Param (M) ↓	FLOPS (G) ↓	Speed (FPS) ↑	ECSSD	DUT-OMRON	DUTS-TE	HKU-IS	THUR15K
MobileNet * [30]	4.27	2.2	295.8	0.829	0.656	0.696	0.816	0.675
MobileNetV2 * [31]	2.37	0.8	446.2	0.820	0.651	0.676	0.799	0.660
ShuffleNet * [32]	1.80	0.7	406.9	0.831	0.667	0.709	0.820	0.683
ShuffleNetV2 * [33]	1.60	**0.5**	**452.5**	0.812	0.637	0.665	0.788	0.652
ICNet [82]	6.70	6.3	75.1	0.838	0.669	0.694	0.812	0.668
BiSeNet R18 [83]	13.48	25.0	120.5	0.829	0.648	0.699	0.819	0.675
BiSeNet X39 [83]	1.84	7.3	165.8	0.802	0.632	0.652	0.784	0.641
DFANet [84]	1.83	1.7	91.4	0.799	0.627	0.652	0.778	0.639
HVPNet [19]	1.23	1.1	333.2	0.854	**0.699**	0.730	**0.839**	**0.696**
SAMNet [16]	1.33	**0.5**	343.2	0.855	**0.699**	0.729	0.837	0.693
CoSOV1Net (OURS)	**1.14**	1.4	211.2	**0.861**	0.696	**0.731**	0.834	0.688

* SAMNet, where the encoder is replaced by this backbone.

**Table 7:** Our proposed model (CoSOV1Net)’s ranking with respect to existing salient object detection [# Param: number of parameters, ↑: great is the best, ↓: small is the best].

Measure	# Param (M) ↓	FLOPS (G) ↓	Speed (FPS) ↑	ECSSD	DUT-OMRON	DUTS-TE	HKU-IS	THUR15K
$F_{\beta }$	1st	1st	1st	6th	7th	9th	11th	11th
MAE	1st	1st	1st	10th	10th	11th	11th	10th
$F_{\beta }^{\omega }$	1st	1st	1st	11th	9th	11th	15th	11th

**Table 8:** Our proposed model (CoSOV1Net)’s ranking with respect to lightweight salient object-detection models [# Param: number of parameters, ↑: great is the best, ↓: small is the best].

Measure	# Param (M) ↓	FLOPS (G) ↓	Speed (FPS) ↑	ECSSD	DUT-OMRON	DUTS-TE	HKU-IS	THUR15K
$F_{\beta }$	1st	6th	7th	1st	3rd	3rd	3rd	4th
MAE	1st	6th	7th	1st	1st	1st	1st	2nd
$F_{\beta }^{\omega }$	1st	6th	7th	1st	3rd	1st	3rd	3rd

## Data Availability

The ECSSD dataset is available at 09 June 2023. https://www.cse.cuhk.edu.hk/leojia/projects/hsaliency/dataset.html. The DUT-OMRON dataset is available at 09 June 2023. http://saliencydetection.net/dut-omron/. The DUTS-TR and DUTS-TE datasets are available at 09 June 2023 http://saliencydetection.net/duts/. The HKU-IS dataset is available at 09 June 2023. https://i.cs.hku.hk/~yzyu/research/deep_saliency.html. The THUR15K dataset is available at 09 June 2023. https://mmcheng.net/code-data/. The SAMNet [16] model’s datasets are available at 09 June 2023. https://github.com/yun-liu/FastSaliency/tree/master/SaliencyMaps/SAMNet. The HVPNet [19] model’s datasets are available at 09 June 2023. https://github.com/yun-liu/FastSaliency/tree/master/SaliencyMaps/HVPNet. Our data results (CoSOV1Net saliency maps) are available at 16 July 2023. http://www.iro.umontreal.ca/~mignotte/ResearchMaterial/COSOV1NET-Data/.
